# An implementation science approach to determine the barriers and facilitators to hepatitis C virus testing in English remand prisons: a mixed-methods study

**DOI:** 10.1136/bmjopen-2024-092965

**Published:** 2025-10-29

**Authors:** Kathryn Jack, William Irving, Zoe Rose, Brian James Thomson

**Affiliations:** 1 Institute of Care Excellence, Nottingham University Hospitals NHS Trust, Nottingham, UK; 2 School of Health Sciences, Centre for Evidence Based Healthcare, University of Nottingham, Nottingham, UK; 3 School of Life Sciences, University of Nottingham, Nottingham, UK; 4 Department of Microbiology, Nottingham University Hospitals NHS Trust, Nottingham, UK; 5 Acute Medicine, Nottingham University Hospitals NHS Trust, Nottingham, UK

**Keywords:** Delivery of Health Care, Integrated, Hepatology, Nursing Care, Patient Care Management, Prisons, QUALITATIVE RESEARCH

## Abstract

**Abstract:**

**Background:**

Testing rates for hepatitis C virus (HCV) of new prison entrants vary considerably between prisons, with particularly low rates in category B male remand prisons. Improvement in testing rates will require an understanding of the underlying reasons.

**Objectives:**

To investigate the rates and uptake of testing for HCV in new entrants to three category B prisons in England and to use an implementation science framework to analyse the facilitators and barriers to meeting national standards for HCV testing in a prison healthcare environment.

**Methods:**

**Design:**

This mixed-methods non-interventional study collated three data sets: anti-HCV testing uptake in prisons, plus data on the prior location of each individual (transfer from another prison or community) and their length of stay; a questionnaire designed to identify reasons for decline of a test administered to people in prison (PIP) who refused testing; qualitative interviews with key stakeholders in the process of prison HCV testing, with analysis based on the Consolidated Framework for Implementation Research (CFIR) to enable identification of barriers and facilitators to testing.

**Setting:**

This study was conducted in the East Midlands region of England.

**Participants:**

Data were obtained from three category B male remand prisons.

**Results:**

Primary outcome measures: This descriptive study sought to understand factors that influence anti-HCV test uptake in three English remand prisons. The selected prisons serve a combined population of 2.3 million and have the capacity to accommodate a total of 2030 prisoners. The testing rates within 4 weeks of arrival in the three prisons over a 12-month study period (March 2022–March 2023) were 17.2%, 28.3% and 42.5%. PIP were more likely to be tested if they arrived from the community compared with interprison transfer (39.13% vs 29.5%). Testing uptake rates increased with length of prison stay (12.4%, 33.6% and 40.7% for stays of 0–7, 15–21 and >28 days, respectively). The most common reasons for not accepting a test were a lack of interest and not wanting to be retested. 13 semistructured interviews revealed 21 barriers and 9 facilitators to testing, summarised in 5 overarching themes: misunderstanding of the concept of opt-out testing; nurses not meeting performance targets due to competing priorities; prison regime hampering healthcare delivery; absence of a specifically appointed co-ordinator who is held to account; incentivising nurses to test and PIP to accept testing.

**Conclusions:**

The rates of testing for HCV in three category B male remand prisons were far below national standards. Key recommendations to improve testing rates, based on the CFIR analysis are (1) to appoint a dedicated senior healthcare staff member who combines responsibility, accountability and authority to proactively oversee testing and ongoing referral processes; (2) to reintroduce an education programme for prison healthcare teams to teach about HCV, cirrhosis and how to deliver ‘opt-out’ conversations and respond to typical responses and (3) to adopt more widely the strategy already shown to be successful in increasing test uptake by the Hepatitis C Trust HITT programme and offer simple incentives.

**STRENGTHS AND LIMITATIONS OF THIS STUDY**

- This is a National Health Service and His Majesty’s Prison and Probation Service research ethics-approved study to address the reasons for the very low rates of anti-hepatitis C virus (HCV) testing rates in English category B male remand (short-stay) prisons.
- The prison estate is a critical environment for research into key public health questions, but unpredictable shortages of staff time and availability and security concerns compromise both the delivery of healthcare and the conduct of clinical research within the prison.
- Our study is underpinned by an Implementation Science theoretical framework, the Consolidated Framework for Implementation Research (CFIR), which is configured to identify barriers and facilitators to anti-HCV testing in a context-specific manner and, therefore, uniquely well suited to research in a prison environment.
- Qualitative interviews were conducted with key staff members and the data were analysed deductively against the CFIR a priori framework, enabling key barriers and facilitators of testing to be identified and inform future healthcare strategies.
- The data collection is limited to the experiences of implementing opt-out anti-HCV testing in three category B male-only remand prisons within a single geographical region.

## Background

 Viral hepatitis infections are a major driver of premature mortality due to end-stage liver disease and hepatocellular carcinoma. In 2016, the WHO reported that global mortality attributable to viral hepatitis was 1.4 million annually as a consequence of both acute infections and progression to end-stage liver disease and hepatocellular carcinoma due to chronic infection with hepatitis C virus (HCV) and hepatitis B virus.^1^ This mortality is comparable to that caused by malaria and HIV. In response, the WHO launched a global strategy to eliminate viral hepatitis as a public health concern by 2030. Key targets were to diagnose 90% of people with chronic infections, to treat 80% who were clinically eligible, to reduce new infections by 90%, and to reduce mortality by 65%.^1^

The recent availability of safe and effective oral direct acting antiviral drugs has resulted in a 51.6% reduction in the estimated HCV prevalence in England from n=129 400 in 2019 to n=62 000 in 2022 and a reduction in mortality of 35% between 2015 and 2022. These figures exceed the WHO interim mortality reduction target of 10% by 2020.^2^

The predominant risk factor for ongoing HCV transmission in the UK is the sharing of contaminated illicit drug injecting paraphernalia. This activity is prevalent in UK prisons and the prison estate consequently harbours a higher proportion of HVC positive individuals than the general community. Current estimates of HCV prevalence indicate that 6% of people in prison (PIP) are anti-HCV positive in contrast to 0.7% in the general population.^2^ Therefore, rigorous control of HCV in UK prisons is critically important to meeting the 2030 targets set by the WHO. In pursuit of this goal, an opt-out approach to blood-borne virus testing in prisons was introduced in England in 2014 and became operational in all establishments by 2018.^3^ The test uptake target was 75%, with the focus on HCV.^4^ More recently, this national elimination strategy has been configured as a summation of more manageable targets such as individual drug treatment services or prisons, with the goal reframed as microelimination.^5^ Prison HCV microelimination status is recognised by achieving three goals: testing a minimum of 95% of the prison population for HCV in the previous 12 months; treating a minimum of 90% of those who have tested positive for HCV RNA and having quarterly reviews of testing and treatment uptake.^6^ The HCV test uptake in prisons in England is now increasing, with 71% of PIP tested within 2 weeks of their arrival.^7^ However, there remains considerable variation between prisons, and many category B remand establishments, which typically accommodate men who are awaiting sentencing, serving a very short sentence, nearing the completion of a long sentence and/or being prepared for release in their home town, appear to have greater challenges in achieving and sustaining the test uptake targets.^8^ Key determining factors behind the lower rates of testing reported by prison nurses in remand prisons centre on the higher numbers of PIP admitted and short duration of stay in contrast with establishments that accommodate people who are sentenced, along with the perspective that many PIP refuse to be tested.^8^,^10^

Against this background, the aim of this mixed-methods study is to investigate in detail rates and timing of HCV testing in selected category B establishments, and to identify barriers to sustainable achievement of national targets using implementation science.

## Methods

### Study design

This mixed-methods study comprised three data sets: (1) anti-HCV testing uptake data derived from three category B remand male prisons in the East Midlands; (2) questionnaire-based data from PIP who refused an anti-HCV test and (3) qualitative interviews, undertaken through an a priori phenomenology lens with 13 key stakeholders.

### Participating sites

Three sites were selected from the complement of 30 category B Remand prisons in England and Wales. All the selected sites are in the East Midlands of England, which is the same geographical location as the authors’ place of work and all have their healthcare managed by the National Health Service (NHS). Further, the three prisons selected contained sites representative of both historically low and historically high testing rates.

### Patient and public involvement

Research within UK Prisons is strictly regulated and governed by Her Majesty’s Prison and Probation Service National Research Committee. Under these restrictions, it is not possible to involve PIP in the design or conduct of the study or in dissemination of results. The outcomes of this study will be shared with UK liver disease and HCV charities for circulation within their networks.

### Study rationale

#### Anti-HCV test uptake data, length of stay and prior location

The primary purpose of our study was to assess progress towards the national target of testing 75% of new prison entrants within a 2-week period and determine the factors which impact on testing rates. In order to address whether testing of entrants after this period could increase numbers tested, we elected to extend our data collection to 4 weeks. Known HCV positivity in PIP who were already inmates was collated but was not linked to date of testing. We recorded the following four variables from each of the prisons for 52 weeks between 21 March 2022 and 20 March 2023: the number of new entrants to each of the three establishments per week; the date of their anti-HCV test; their prior location (categorised either as admitted from another prison or from the community); and their length of stay. An anti-HCV test was recorded if this occurred within the 4 weeks following the week of arrival. The length of stay data were grouped into the numbers of PIP who remained for 0–7, 8–14, 15–21, 22–28 and 29 or more days. Data on the weekly numbers of new entrants to each of the three prisons and anti-HCV test uptake were obtained from the prisons’ electronic health records system by the research delivery nurse team and prison nurses. The length of stay and prior location is held on the electronic prison management system and can legally only be accessed by prison employees. These data were therefore obtained by the prison nurses. The number of prison entrants and test uptake data were obtained for 52 weeks. Length of stay and prior location metrics were collected from prison #1 for 3 weeks, prison #2 for 28 weeks and prison #3 for 28 weeks—a total of 59 prison weeks. As noted above, access to these variables is wholly dependent on the availability of prison staff and the prison security regime, which unavoidably limited data collection. A χ^2^ test of independence was performed using SPSS V.29 to examine the relationships between test uptake, length of stay and location prior to prison arrival.

#### Rationale for anti-HCV test refusal

During the same time frame, PIP who had not been tested for anti-HCV within 4 weeks of their arrival at the prison were invited to complete a questionnaire (see online supplemental file 1). The questionnaire was designed by the study team and comprised three groups of questions: first to ascertain if there was a medical reason why it was not appropriate to offer a test, such as a diagnosis of dementia or acute psychosis; second to ascertain the PIP’s reasons for refusal which included: a belief they were not at risk; having been tested recently elsewhere; already known to be HCV positive; needle phobia; not interested; had never taken intravenous drugs; asked why they should be tested. The third section was to record if there had been any prison security regime related reason that would have prevented PIP from accessing a routine healthcare appointment for testing. The questionnaires were administered by prison nurses during the clinic appointment when PIPs were offered a test. The nurse and PIP worked together to ensure that all applicable boxes were ticked, ensuring a very high completion rate of the questionnaire. Successful completion of this study aim was also dependent on availability of prison staff and time within the prison security regime. Verbal consent from the PIP to collect these data was obtained, as per the NHS and NRC research ethics committee approvals.

#### Semistructured interviews with key prison healthcare personnel

The qualitative interviews were underpinned by implementation science methodology, defined as ‘the scientific study of methods to promote the systematic uptake of research findings and other evidence-based practices into routine practice, and hence, to improve the quality and effectiveness of health services’.^11^ The implementation of an evidence-based practice intervention, in this instance being to test people entering prisons for anti-HCV, frequently fails due to the influence of local contextual factors.^12^ The use of a theory-driven determinant framework can support the identification of features that affect the successful implementation of an intervention.^12^ The Consolidated Framework for Implementation Research (CFIR) comprises five contextual domains containing 39 constructs (terms and conditions) that enable clear identification and articulation of the barriers and facilitators to implementing the intervention in question.^12^ The CFIR questionnaire and further details of how this framework was constructed can be found in the online supplemental file 2. Key prison healthcare professionals involved in direct and indirect implementation of the anti-HCV opt-out testing process from the three prisons were invited by telephone and letter to participate in an audio-recorded semistructured interview and 13 staff, 3 of whom were previously known to the interviewer, provided written consent. Seven staff who were invited to participate declined due to lack of interest. This final number recruited was determined by the principles of data saturation and information power,^13^ whereby participants are interviewed until there are no further insights, patterns or themes identifiable. The interviews were conducted via the telephone by the lead author, who is a female postdoctoral nurse and qualitative researcher, has undertaken previous prison research and has more than 20 years experience in HCV service delivery and qualitative research. The mean interview duration was 40 min and no additional field notes were taken. To preserve anonymity among a small community of staff employed in these locations, details of their characteristics and job roles have been omitted from this paper. The interview guide was aligned to the five domains containing 39 constructs of the CFIR, namely: Intervention characteristics, Outer setting (defined as prison healthcare in general), Inner setting (defined as the prison in which the participant was employed), Characteristics of the individuals and the Implementation process.^14 15^ The audio-recorded interviews were anonymised and professionally transcribed. The data were analysed deductively following a process of data familiarisation and checking for fidelity against the audio recordings, initial coding and aligning data to the constructs and domains in the CFIR tool.^14^ Relevant constructs in each domain were assigned as either a barrier or facilitator (actual or proposed) to the anti-HCV testing implementation process. The textual data were managed manually using paper and highlighter pens, and an Excel spreadsheet. All 13 interview transcripts were analysed by one author (KJ) and seven were reviewed by a second author (ZR) to reduce interpretation bias. Data interpretations and differing perspectives were discussed and resolved without a need for a third person. The Consolidated criteria for Reporting Qualitative research and Strengthening the Reporting of Observational Studies in Epidemiology reporting guidelines were used to support this study (see onlinesupplemental files 3  4).

### Findings

#### Anti-HCV test uptake data, including length of stay and prior location

Anonymised anti-HCV test uptake was available for the full 52 weeks’ timeframe. The cumulative testing rates for all three prisons are illustrated in table 1. Only 26.1% of PIP were tested for HCV within 2 weeks of prison entry, far below the national target of 75%. Further testing rates in each prison were highly variable. Prisons #1, #2 and #3 tested 17.2% (n=594/3456), 28.3% (n=590/2081) and 42.5% (n=558/1307), respectively, within 4 weeks following the week of their arrival.

**Table 1:** Length of stay and anti-HCV test uptake n=1836 (data collected in prison #1 for 3 weeks and in prisons #2 and #3 for 28 weeks duration between 3 March 2022 to 26 September 2022)

Duration in prison	n=PIP	%=PIP	n=tested for anti-HCV	%= tested for anti-HCV
0–7 days	186	10.1	23	12.4
8–14 days	161	8.8	42	26.1
15–21 days	107	5.8	36	33.6
22–28 days	114	6.2	47	41.2
29 days or more	1268	69.1	516	40.7
Total	1836		664	

HCV, hepatitis C virus; PIP, people in prison.

Information on length of stay and prior location was obtained for a subset of PIP by prison nurses. Data were available for 28 weeks from prisons #2 and #3 individually (53.8% of the test uptake timeframe), and for 3 weeks from prison #1 (1.6% of the test uptake time frame), giving a detailed data set from 1836 PIP admitted during a combined period of 59 weeks. Limitations imposed on collecting this data are set out in the Methods section above. Overall rates of anti-HCV testing in this subgroup were 36.2% (n=664/1836). 63.6% (n=1168/1836) of individuals had been admitted from the community, of whom 39.13% (n=457/1168) were tested; 36.4% (n=668/1836) had been admitted by inter-prison transfer, of whom 29.5% (n=197/668) were tested (p≤0.001).

Although category B remand prisons have a high turnover, our data show that 69.1% (n=1268/1836) of the PIP remained in the prison for 29 days or more. Table 1 shows that test uptake rates increased as duration of stay increased (p≤0.001), which confirms the anecdotal reports by prison nurses that testing continues beyond the 2-week data collection time frame adopted by the UK Health Security Agency (UKHSA).^7^

### Reasons for refusal of an HCV test

Reasons for anti-HCV test refusal were obtained by questionnaire during a 43-week period between 21 March 2022 and 15 January 2023. n=5469 people were admitted to the three prisons in that time, of whom only 25.73% (n=1407/5469) were tested for anti-HCV. The number of PIP not tested therefore vastly exceeded the study teams’ predictions which had been based on the available literature at the time of designing the study. It was beyond the capacity of the study to establish the reasons why n=4062 PIP refused a test. Data on the reasons for not being tested were sampled using an unselected group of n=166 PIP and are set out in table 2. The principal limitation on this data collection was constraints on the availability and time of prison nurses. Two PIPs had been assessed by the registered nurse on admission as not having sufficient mental capacity to consent to a test (1×acute psychosis and 1×severe attention-deficit-hyperactivity-disorder). There were no prison regime-related reasons to have prevented access to a test reported by this sample. Our findings confirmed consistent anecdotal feedback from prison healthcare staff that the dominant reasons for refusing an anti-HCV test are a lack of interest and not wanting to be retested.

**Table 2:** Rationale for declining an anti-HCV test

Prison	Prison #1 n=	Prison #3 n=	Total n (%)
Participants	64	102	166
PIP reports a recent anti-HCV test	17	23	40 (24.1)
Known anti-HCV positive	1	2	3 (1.8)
Needle-phobic	1	1	2 (1.2)
‘I am not interested’	35	75	110 (66.3)
‘I am not a drug user’	25	0	25 (15.1)
‘Why should I?’	32	0	32 (19.3)
Refused to give a reason for refusal	0	1	1 (0.6)

HCV, hepatitis C virus; PIP, person/people in prison.

### Qualitative interview data

13 semistructured interviews were conducted with key healthcare professionals (doctors and nurses) across the three prisons. The participants’ roles were in primary care (n=9), substance misuse services (n=3), and both (n=1). Table 3 shows the domains and number of constructs which were identified as either or both an anti-HCV testing barrier or facilitator.

**Table 3:** Consolidated Framework for Implementation Research domains and number of constructs identified as barriers and facilitators to implementing opt-out anti-HCV testing

Domain	Domain definition	N=available constructs	N=constructs as barriers	N=constructs as facilitators
Intervention Characteristics	Opt-out anti-HCV testing	8	5	1
Outer setting	General prison environments	4	4	1
Inner setting	The prison where the participant works	14	8	4
Characteristics of individuals	Individual experience and views	5	2	0
Process	Implementation process	8	2	3

HCV, hepatitis C virus.

Healthcare staff who participated in the qualitative semistructured interviews identified a number of barriers and facilitators to the achievement of high levels of HCV testing in prison that can be summarised in these five overarching themes: misunderstanding of the concept of opt-out testing; nurses not meeting performance targets due to competing priorities; prison regime hampering healthcare delivery; absence of a specifically appointed co-ordinator who is held to account; incentivising nurses to test and PIP to accept testing. Tables4  5 identify these barriers and facilitators, accompanied by a summary of the responses expressed. More comprehensive versions of tables4  5 with verbatim illustrative quotes are available as supplementary material (see online supplemental file 5). Of the 39 available CFIR constructs, there are 21 barriers and nine facilitators.

**Table 4:** Consolidated Framework for Implementation Research (CFIR) barriers to implementing anti-HCV testing in prisons

CFIR domain and construct	Construct descriptor	Summary of responses
I.A	Knowledge about intervention source	Only one participant knew about the background to opt-out testing
I.B	Strength and quality of Intervention evidence	No participants were aware of any research evidence behind the opt-out approach. Introduced ‘top down’
I.E	Was the intervention piloted?	Not piloted in all prisons to identify local issues
I.F	Intervention complexity and understanding definition	Opt-out testing and DBST testing considered to be the same by the majority, most responses focused on the concept of choice.
I.G	Intervention packaging/training	Initial training delivered by written material, not in person, agency nurses not included, training focused on the DBST not opt-out approach
II.B	Knowledge of the patient’s needs and resources	When people first enter a prison, the reception process is too busy for the PIP to think about a HCV test, especially in a remand prison. Some PIP feel they don’t need a test because they have never injected drugs, some fear being found to be HCV positive so decline a test, testing fatigue occurs with repeated testing for frequent reoffenders, and some PIP find it hard to trust people including nurses.
II.B	Cosmopolitan—opportunities to meet other nurses to discuss the intervention	Registered nurses don’t get the chance to meet nurses from other prisons in the region or nationally to share best practice and problem solve unless they are a matron (a managerial role), and attendance can be determined by staffing levels. Liaising with hospital specialist nurses is very helpful.
II.C	Peer pressure—how this can influence opt-out HCV testing	One participant paraphrased negative comments overheard about the value of opt-out HCV testing in prisons. These views constitute negative peer pressure that could discourage testing.
II.D	External policies and incentives	Linked to A1 and A2. Participants not aware of the policy to test 75% of PIP on arrival. Nationally written prison regime policies are enforced robustly by prison officers, which impacts on opportunities for healthcare provision. National Health Service England/public health policy for opt-out HCV testing is criticised because ‘outsiders’ don't understand “prison life”.
III.A	Structural characteristics	The lack of space in the reception area and on the wings for healthcare provision was reported by most participants. Some rooms were structurally dilapidated. Most nurses identified there was insufficient space to do testing.
III.B	Networks and communications	Prisons are hierarchical organisations. Healthcare roles don’t overlap; for example, substance misuse, mental health and primary care nurses all have very distinct remits. Successes are dependent on working relationships with prison staff.
III.C	Work culture	Many prison officers are perceived to be uncaring, and not all are supportive of preventative healthcare activity. There was some evidence of a poor work culture among the nurses too.
III.D	Implementation climate	Negativity was identified among some participants, with low staff morale and a reluctance to work observed in some colleagues. This results in some nurses being uninterested in HCV and all BBV testing and a view held by some that there is no point in testing because it takes too long to get results back.
III.D.3	Relative priority of the intervention compared with others	The majority of participants’ comments were in this domain. The priority in reception is to get the PIP in and processed, assess for medicines needed and acute issues to respond to for their first night, to assess for suicide risk or self-harm and avoid deaths. Other healthcare priorities include: wound care/dressings, emergency responses, cancers, long term conditions, vaccinations for example, influenza/Covid. During their sentence, PIP may be more interested in addressing other health issues, for example, dental care, and may also be concerned with managing threats from gang members, bullying and being a vulnerable prisoner. The prison regime is a significant competing priority.
III.D.9	Goals and feedback	There is limited feedback given to nurses about process or achievements, numbers tested or the outcomes. Sometimes feedback about low test uptake is used punitively with limited recognition that PIP refuse tests.
III.E.1	Leadership engagement	Prison leadership: healthcare staff are keen to make changes but there can be resistance from some prison officers because it can mean extra work for prison officers to escort PIP to healthcare. Prison Governors can block HCV Trust peer worker access. Healthcare leadership: Limited or sporadic encouragement to achieve higher rates of anti-HCV testing from senior leadership teams was observed by a minority of participants.
III.E.2	Available resources	Agency nurses make up the number of staff needed each shift but they cannot lead implementation projects. There are insufficient nurses, and it is difficult to recruit nurses to work in a prison in the context of a national shortage. There are often insufficient prison officers too, and this combined lack of staff is a significant resource gap.
IV.B	Staff self-efficacy	Individual staff self-efficacy is variable. A few participants report that some nurses are apathetic towards HCV testing and that this is generally driven by staff who are interested rather than it being an embedded process.
IV.E	Other personal attributes	Most prison nurses won’t have clinical experience of looking after people with liver disease. However, one participant had looked after people with decompensated cirrhosis.
V.B.2	Formally appointed Internal opinion leaders	Unlike the prisons whose healthcare services are run by Patient Partnership Group, there are no formally appointed leaders employed by the three prisons. This absence of a key individual to lead this aspect of service delivery was observed by a minority of the participants.
V.B.3	Champions	Champions may be officially appointed, such as the national group of hepatitis C Trust employed peer workers who are able to support education and testing. Other Champions may be current PIP who are influential among their peers and willing to support others. However, PIP don’t always engage with Champions because of trust and confidentiality, and they may have an ulterior motive for seeking a role where they can move more freely in the prison. Hard to find the right person to be a Champion in a cat B because of the high turnover. One prison would like to have HCV Trust prison peer workers, but the Governor has refused.

BBV, bloodborne virus; DBST, dried blood spot testing; HCV+, hepatitis C virus; PIP, person/people in prison.

**Table 5:** Consolidated Framework for Implementation Research (CFIR) facilitators to implementing anti-HCV testing in prisons

CFIR domain and construct	Construct descriptor	Summary of responses
I.D	Adaptability—changes made or suggested to improve test uptake	Changing the timing to subsequent healthcare appointments, extending substance misuse nurses’ remit to test, enabling prison and community electronic health records to be compatible to aid communication. Prison officers to tell the PIP that testing is routine.
II.B	Cosmopolitan—opportunities to meet other nurses to discuss the intervention	Matrons (band 7s) meet regularly with other band 7s in their region, and there are good links with hospital HCV nurses who share information and support. It would be helpful for the staff nurses to have external peer support too.
III.A	Structural characteristics	Newer prisons have purpose-built healthcare space which facilitates improved care delivery.
III.D.4	Incentives and rewards	Incentivising the PIP is highly effective, for example, shopping vouchers to have on release, phone credit, puzzle books, extra gym visits. Financial incentives for nurses to work in prisons were suggested.
III.D.5	Goals and feedback	Seeing people cured is motivating. Important to give feedback about test uptake that recognises different prison contexts.
III.E.3	Access to knowledge and information	Increase provision of teaching nurses about the opt-out approach, test methods and HCV (and other BBVs). Include as part of the formal staff induction process.
V.A	Planning for intervention implementation	External support from the NHS Trust’s Quality Improvement team using the Plan, Do, See, Act methodology. Nurses monitored the day-to-day testing data (same as Patient Partnership Group).
V.B.2	Formally appointed Internal opinion leaders	Noted by the research team that the prison healthcare departments run by the Patient Practice Group also employ a regional HCV lead nurse to oversee the HCV testing programme. The authors and one participant suggest that a similar NHS role would be advantageous in all category B local prisons.
V.B.4	External change agents	HCV Trust employed prison peer workers are valued. Outside help from Hep C Trust who deliver the High Intensity Test and Treat events is welcomed.

BBV, bloodborne virus; HCV, hepatitis C virus; NHS, National Health Service; PIP, person/people in prison.

## Discussion

In this study, we present the results from our investigation of rates of testing for HCV in three category B Prisons with healthcare services provided by the NHS. The first and most striking observation is that testing rates in these prison environments were very far below expected national targets, confirming the challenge of achieving high rates of HCV testing in a category B local prison. These results also support recent data in Welsh prisons.^9^ Of particular concern is the finding that just 39.13% (n=457/1168) of PIP admitted from the community were tested. Although this figure is higher than for PIP admitted by interprison transfer, the characteristics of the population admitted to remand prisons include particular risk of bloodborne virus acquisition due to high rates of injecting drug use prior to admission.^16 17^ While unsatisfactory, the figure of 39.13% is a substantial improvement from data in our previous study where only 9.7% (n=1140/11 768) were tested.^8^ While there is clear evidence of a high turnover of PIP in the test prisons, with 10.3% staying for less than 7 days, the majority of PIP in this study (68.9%) were still in the same remand prison for more than 4 weeks. Our data show that opportunities exist to roll out testing to PIP who have not been tested in the initial 2-week period specified by UKHSA and that this approach may be constructive, particularly in prisons where prison staff time and availability is particularly challenged.

The questionnaire to PIP who had refused a test was administered to a subset of 166 individuals from a far higher proportion of inmates refusing a test than anticipated. This is likely to be a consequence of pressures on staff time and security requirements for an exercise that required additional nurse time to complete. The very high denominator population who were not tested is also likely to be a function of other limitations in a category B prison environment. It is, for example, not possible from prison records to differentiate between prisoners who refused and those who were not offered a test. Nonetheless, our results do confirm in 166 individuals that by far the dominant reason for refusing a test is lack of interest, and this has important implications for prisoner and staff education on risks of HCV infection.

A thematic analysis of our data indicates that barriers and facilitators to test uptake generally lie in multiple domains in the CFIR, in keeping with findings from a Canadian study in remand prisons,^18^ although in our study none of the characteristics of staff delivering the intervention (Domain IV: Characteristics of Individuals) emerge as important variables.

In common with our previous study,^8^ there was persistent evidence of misunderstanding of the concept of opt-out testing (Domain I.F in table 5). The opt-out approach to testing was frequently conflated with the test method, which was to obtain a dried blood spot sample (CFIR I.F-Complexity of intervention). Although education is reportedly delivered on this topic, the majority of staff interviewed were not familiar with either the rationale or the evidence base for this approach. The educational programmes in the test prisons, therefore, clearly do not deliver the information and understanding needed by the prison nurses to operate an opt-out testing policy (CFIR I.G-Intervention packaging/training), despite this being recognised as an intervention enabling factor elsewhere.^19 20^ A renewed and ongoing (to accommodate staff turnover) programme of education about cirrhosis, HCV, test methods, approaches to discussing the need for testing and treatment would address this and other CFIR barriers identified in the Intervention Characteristic domains (I.A-Intervention source, I.B-Evidence strength and quality, I.F-Intervention complexity and understanding definition, and I.G-Intervention packaging/training). A proposed facilitator was to enable more prison nurses to have opportunities to meet colleagues from neighbouring establishments, rather than just the matrons (CFIR II.B-Cosmopolitan). Participants additionally suggested that discussing HCV and sharing best practices and problem-solving with colleagues would be a supportive action. Many nurses interviewed expressed frustration that their category B remand prisons, and thus PIP behaviours, were being directly compared with prisons housing sentenced individuals who had overcome the disruption of initial admission and would be in one place for a longer duration. The competing clinical priorities leading to the de-prioritisation of anti-HCV testing, which have been a very evident theme in our study, have also been noted elsewhere.^20^ The consequent frustration and demoralisation experienced by nurses who were criticised for underachieving in a clinical context is unlikely to enable success.

The majority of barriers to testing lie in domain III, the ‘Inner setting’, which in this study refers to the prison contextual environments. The staff interviews revealed multiple examples of the prison regime hampering healthcare delivery. The ‘bus to bed’ time frame refers to the national policy mandating that people entering a prison are held in the reception area for the shortest time possible.^21^ This adds to the pressure placed on prison nurses to complete the required health checks in the shortest possible time, while assessing for physical and mental healthcare needs that could result in harm or death if not immediately addressed, including a risk of suicide (see domains, ie, II.A and III.D.3 in table 5). In addition, healthcare visits may be cut short or PIP not collected to attend appointments because of the requirement to lock up the PIP at certain times. The importance of acknowledging the different context of a prison in contrast to hospital or community environments when constructing and delivering healthcare interventions has been highlighted in other studies.^22^ ^23 24^ Many participants discussed the difficulties in delivering the anti-HCV testing policy when PIP arrived, or early in their stay, because of the psychological stress or drug withdrawal symptoms experienced at this time. Improving opportunities to deliver healthcare will support PIP need at this time (CFIR II.A-Patients needs and resources).

Another key barrier was the absence of a recognisable individual with responsibility for delivery of all aspects of the testing policy. There is a need to appoint a dedicated staff member to systematically oversee the test uptake of each person admitted to prison and who is accountable to both healthcare commissioners and the hospital hepatitis operational delivery network lead (see CFIR III.E.1-Leadership engagement and E4-Internally appointed key opinion leader). The postholder would be in a position to navigate the competing priorities (CFIR III.D.3-Relative priority), negotiate the adaptations needed to access PIP at different times in a remand prison (CFIR I.D-Adaptability), and optimise the use of staff and healthcare rooms (CFIR III.E.2-Available resources). Further, this staff member would also be able to hold the prison nurses to account and promote consistent intervention delivery (CFIR IV.B-Staff efficacy).

A possible facilitator raised by the interviewees was the use of incentivisation. There are multiple opportunities to incentivise both staff and PIP to increase testing activity and PIP acceptance. The data in the CFIR domain III.D.5, Goals and feedback, identifies a need to provide appropriate context-dependent test uptake goals. The majority of PIP in our study who gave a rationale for declining a test stated they were not interested (see table 2), so giving an incentive may overcome this response. Offering incentives to PIP to accept a healthcare intervention is ordinarily not permitted by the prison service because it is considered to be a coercive action and could also lead to the individual placing demands on the staff to bring additional goods including contraband into the establishment. However, the High Intensity Test and Treat (HITT) hepatitis C testing programme managed by the Hepatitis C Trust (CFIR V.B.4-External change agents), which sends a team of staff to test all PIP over 3–5 days, routinely offers incentives such as confectionery and this contributes to the high test uptake achieved during these events. Incentives (CFIR I.D-Adaptability) can encourage PIP to reconsider priorities (CFIR III.D.3-Relative priorities, table 4 Rationale for declining a test—‘recently tested’ and ‘not interested’) when they are considering how to spend their time when not confined to their cell. Incentives such as phone credits, toothbrushes or puzzle books may be acceptable to prison and healthcare service leaders alike.

Overall, the current financial and staff recruitment challenges experienced by both the prison and healthcare service are combining to render the implementation of critical public health interventions extraordinarily difficult. While this study team is mindful of these challenges and recognises that prisons are a stressed system, our data also provide an evidence base for how healthcare and prison services can work together to optimise the delivery of healthcare within the current financial and staffing restrictions.

### Limitations

This study is subject to a number of limitations. The CFIR interviews are from three category B male remand prisons managed by the NHS in a single geographical region in England. Although our study was large (a denominator population of almost 7000 PIP), further research in a wider geographical area and a larger number of staff interviewed may reveal different data. The key limitation throughout our study, however, was that non-prison staff are not permitted to make direct contact with prisoners or access data kept on prison information systems. Many key aspects of our research, and the success of the prison healthcare systems being studied, are a function of the time and availability of the prison staff, which were often and unavoidably compromised. Our study, however, uses a ‘real world’ and context-dependent methodology, and these restrictions are a necessary part of our analysis.

### Conclusions

Our study has confirmed previous concerns that anti-HCV test uptake in some male category B remand prisons managed by the NHS remains far short of national standards. Our study operated in an environment where the availability of prison staff key to both facilitating our study and delivering healthcare was often unavoidably compromised. This enabled us to unpack explanatory findings which were contextual and which identified barriers and facilitators to implementing the opt-out testing intervention in the study prison environment. Our results identify three pragmatic interventions which are potentially deliverable in the majority of prison environments and could facilitate sustainable processes to anti-HCV testing and a reduction in the negative impact of barriers. first, the appointment of a dedicated senior healthcare staff member who has the overall responsibility, accountability and authority to proactively oversee testing and ongoing referral processes. second, to reintroduce an education programme for prison healthcare teams to teach about HCV, cirrhosis and how to deliver ‘opt-out’ conversations and respond to typical responses. Third, to adopt more widely the strategy already shown to be successful in increasing test uptake by the Hepatitis C Trust HITT programme and offer prison nurses and PIP simple incentives such as confectionery. Finally, it is clear that when PIPs are presented with the ability to opt out of a healthcare intervention, a substantial number do so. It may be timely to modify the approach to prison-based HCV testing and reframe the current testing intervention as ‘routine’ rather ‘opt-out’ because this may minimise future uncertainty regarding the importance of ongoing testing for HCV in prisons.

## Supplementary material

10.1136/bmjopen-2024-092965 online supplemental file 1

10.1136/bmjopen-2024-092965 online supplemental file 2

10.1136/bmjopen-2024-092965 online supplemental file 3

10.1136/bmjopen-2024-092965 online supplemental file 4

10.1136/bmjopen-2024-092965 online supplemental file 5

## Data Availability

All data relevant to the study are included in the article or uploaded as supplementary information.
