# Supplementary material for: An implementation science approach to determine the barriers and facilitators to hepatitis C virus testing in English remand prisons: a mixed-methods study
Source: BMJ Open. 2025 Oct 29;15(10):e092965. doi: 10.1136/bmjopen-2024-092965 (PMC12574346; doi:10.1136/bmjopen-2024-092965)
Supplement: online supplemental file 1 [file bmjopen-15-10-s001.docx]

**No Anti-HCV Test (NAT) undertaken record sheet**

**PIP study ID number: …………………………**

**“We are recording the reasons why some people do not get tested for hepatitis C. Your answer will be anonymous and will contribute to research into how to increase the rates of hepatitis C testing in prisons so that it can be eliminated. Thank you”.**

**Capacity to consent yes / no**

1. **Assessed by nurses as not clinically appropriate to offer a hepatitis C test** Yes / No

If Yes:

1. Acute psychosis Yes / No
2. Dementia / Alzheimer’s disease Yes / No
3. Other cause of confusion …………………………………………………………………………………………..
4. Does not understand spoken English Yes / No
5. Verbally / physically violent and not safe for nurse to prolong clinical engagement Yes / No
6. Any other reason ……………………………………………………………………………………………………..
7. **PIP refused testing due to e.g:** (indicate all that apply)

No perceived risk factors Yes / No

Recently tested Yes / No Where…………………………………………………………………….

When………………………………………………………………………

Result …………………………………………………………………….

Known HCV negative Yes / No

Known HCV positive Yes / No

Needle phobic Yes / No

“Not Interested” Yes / No

“I’m not a drug user” Yes / No

“why should I ?” Yes / No

Treated for HCV Yes / No (If yes, details of when/where)

…………………………………………………………………………………………………………………………………………………………………………If yes, any risk factors since treatment yes/ no/ don’t know

Any other reasons …………………………………………………………………………………………………………………………………………

……………………………………………………………………………………………………………………………………………….………………………

1. **Unable to conduct test due to prison regime / other** Yes / No

Details…………………………………………………………………………………………………………………………………………………………

………………………………………………………………………………………………………………………………………………
