# Supplementary material for: An implementation science approach to determine the barriers and facilitators to hepatitis C virus testing in English remand prisons: a mixed-methods study
Source: BMJ Open. 2025 Oct 29;15(10):e092965. doi: 10.1136/bmjopen-2024-092965 (PMC12574346; doi:10.1136/bmjopen-2024-092965)
Supplement: online supplemental file 2 [file bmjopen-15-10-s002.docx]

# Consolidated Framework for Implementation Research (CFIR) Interview Guide

The Consolidated Framework for Implementation Research (CFIR) questionnaire is set within a pragmatic framework constructed following a systematic and comprehensive review of existing theoretical frameworks that enable the translation of research findings into clinical practice [14]. This framework was designed to be used as a qualitative interview guide with constructs that the research teams can personalise for their study according to the specific intervention under review, to enable a transparent evaluation of the barriers and facilitators to successfully implementing the intervention in a specific context [14]. In our study, the CFIR framework adaptations required for application a prison setting were discussed and agreed by the authors KJ, BJT, and WI.

## A. Intervention Characteristics

| ***A. Intervention characteristics (8)*** | ***Questions*** |
| --- | --- |
| ***1.Intervention Source*** | Who developed the opt-out approach to BBV testing |
|  | What is your opinion of this group/person? |
|  | Why is OO BBV testing being implemented in your prison? |
|  | Who decided to implement OO BBV testing being implemented in your prison? |
|  | How was the decision made to implement OO BBV testing? |
|  | (NB: Is the participant conflating OO and DBS ?) |
|  |  |
| ***2. Evidence Strength & Quality*** | What kind of information or evidence are you aware of that shows whether or not the OO BBV testing will work in your prison? |
|  | How does this knowledge affect your perception of OO BBV testing? |
|  | What do the nursing team in your prison think of the intervention? |
|  | What do senior prison staff / governors think of the intervention? |
|  | What kind of evidence or proof is needed about the effectiveness of OO BBV testing to get staff (nurses) on board? |
|  |  |
| ***3. Relative Advantage*** | How does the OO BBV testing compare to the previous testing system? |
|  | What advantages does OO BBV testing have compared to existing programs? |
|  | What disadvantages does OO BBV testing have compared to existing programs? |
|  | Is there another approach to BBV testing that people would rather implement? |
|  | Can you describe that approach? |
|  | Why would people prefer that alternative? |
|  |  |
| ***4. Adaptability*** | What kinds of changes or alterations do you think you will need to make to the OO BBV testing approach so it will work effectively in your setting? |
|  | Do you think *you* will be able to make these changes? Why or why not? |
|  | Who will decide (or what is the process for deciding) whether changes are needed to OO BBV testing so that it works well in your prison? |
|  | How will you know if it is appropriate to make any changes? |
|  | Are there components that should not be altered? |
|  | Which ones should not be altered? |
|  |  |
| ***5. Trialability*** | Was OO BBV testing piloted prior to full-scale implementation? [If Yes] What did this pilot look like? |
|  | Would a pilot have been helpful? |
|  |  |
| ***6. Complexity (of intervention)*** | How complicated is OO BBV testing?  e.g. duration, scope, intricacy and number of steps involved and whether it reflects a clear departure from previous practices. |
|  | What does OO testing mean? Do you have a definition? |
|  |  |
| ***7. Design Quality & Packaging*** | Did you receive any training for OO BBV testing and who from? |
|  | What was / is the quality of the training? |
|  | Do you have access to any support materials or information? How do you access these materials? |
|  | How do these materials affect implementation of OO BBV testing in your setting? |
|  |  |
| ***8. Cost*** | Is there an increased cost associated with OO BBV testing ? |
|  | What costs were considered when deciding to implement OO BBV testing? |

## B. Outer Setting

| ***B. Outer setting (4)*** | ***Questions*** |
| --- | --- |
| ***1. Patient Needs & Resources*** | To what extent are the nurses / nurse managers aware of the BBV testing preferences of the PIP in your prison? |
|  | How do you think the PIP feel about OO BBV? |
|  | To what extent were the preferences of the PIP considered when deciding to implement the intervention?  Can you describe specific examples?  Will OO BBV be altered to meet their needs and preferences? |
|  | How well do you think that OO BBV testing will meet the PIPs’ needs?  In what ways will the OO BBV meet their needs? e.g. improved access to treatment? Reduced wait times? Help with self-management? |
|  | What are the barriers to accessing OO BBV testing faced by PIP? |
|  | Have you asked the prisoners about their experiences with O-O BBV?  What are their perceptions? Specific information? |
|  |  |
| ***2. Cosmopolitan*** | What kind of information exchange do you have with others outside your prison, either about OO BBV, or more generally? |
|  | To what extent do you network with colleagues or people in similar professions/positions outside your setting? How often? |
|  | To what extent does your prison encourage you to network with colleagues outside your own setting? |
|  | Are you able to attend local/national conferences/training? Other venues? |
|  |  |
| ***3. Peer Pressure*** | Can you tell me what you know about any other prisons who have implemented OO BBV testing? Is their experience similar to yours? |
|  | How has this information influenced the decision to implement OO BBV testing? |
|  | To what extent does implementing OO BBV testing provide an advantage for your prison compared to other prisons? |
|  | Is there something about OO BBV testing that would encourage nurse recruitment / retention into your prison? |
|  |  |
| ***4. External policies and incentives*** | What kind of local or national performance targets have influenced the decision to implement OO BBV testing? |
|  | How will OO BBV testing affect your prison’s ability to meet these targets? |
|  | Are there any incentives that have influenced the decision to implement OO BBV testing? |
|  | Does OO BBV testing affect income for your prison? |
|  | Do you receive a specific income for reaching the 50% or 75% targets or are you simply funded to achieve as many as you can of the domains in the section 7a? |

## C. Inner Setting

| ***C. Inner setting (14)*** | ***Questions*** |
| --- | --- |
| ***1. Structural characteristics*** | How does the physical layout of your prison affect the implementation of OO BBV testing? |
|  | How do you work around the challenges? |
|  | What kinds of infrastructure changes will be needed to accommodate OO BBV testing? Who could make those changes? Are they possible to change? |
|  |  |
| ***2. Networks and communication*** | Can you describe your working relationships with your colleagues at this prison? |
|  | Do you get together with colleagues outside of work? |
|  | Can you describe your working relationship with your seniors? |
|  | Do you have regular staff meetings? |
|  | Do you typically attend? / Who typically attends?  How often are the meetings held?  What is a typical agenda? How helpful are these meetings? |
|  | How do you typically find out about new information, such as new initiatives, accomplishments, issues, new staff, and staff departures? |
|  | When you need to get something done or to solve problems, who are your "go-to" people? |
|  |  |
| ***3. Culture*** | Some people characterize culture in terms of four general types. To what extent would you characterize your culture as:  **Team (Clan) Culture (Flexible, Internal Focus):** A friendly workplace where leaders act like mentors, facilitators, and team-builders. There is value placed on long-term development and doing things together.  **Hierarchical (Hierarchy) Culture (Control, Internal Focus):** A structured and formalized workplace where leaders act like coordinators, monitors, and organizers. There is value placed on incremental change and doing things right.  **Entrepreneurial (Adhocracy) Culture (Flexible, External Focus):** A dynamic workplace with leaders that stimulate intervention. There is value placed on breakthroughs and doing things first.  **Rational (Market) Culture (Control, External Focus):** A competitive workplace with leaders like hard drivers, producers, or competitors. There is value placed on short-term performance and doing things fast.  (This question can be open-ended or elicit percentages so that they add up to 100%. e.g., my culture is 50% Team, 40% entrepreneurial, 10% hierarchical). |
|  | How do you think your prison’s culture will affect the implementation of the OO BBV testing? |
|  | To what extent are new ideas embraced and used to make improvements in your prison? |
|  |  |
| ***4. Implementation climate*** | How receptive has this prison’s healthcare team been to implementing OO BBV testing? Why / why not? |
|  |  |
| ***5. Tension for change*** | Is there a strong need for OO BBV testing of the PIP in your prison?  Why or why not? |
|  | Do other nurses see the need for OO BBV testing? |
|  | To what extent did the previous approach fail to meet the testing needs of the PIP in your prison? |
|  | Will OO BBV testing meet these needs? |
|  | How do you/others feel about the subsequent treatment for HCV for PIP? |
|  |  |
| ***6. Compatibility*** | How well does OO BBV testing fit with your personal values and norms? |
|  | How well does OO BBV testing fit with the values and norms of the prison? |
|  | How well does OO BBV testing fit with existing work processes and practices in your prison? |
|  | What are the likely issues or complications that may arise? |
|  |  |
| ***7. Relative priority*** | Are there any other high-priority activities happening in your prison? |
|  | What is the priority of meeting the 75% OO BBV testing targets compared to the other activities that are happening now? |
|  | Does the OO BBV testing target conflict with these priorities? |
|  | Will OO BBV testing help achieve (or relieve pressure related to) these other priorities? |
|  | What kind of pressure are you feeling to achieve the 75% OO BBV test target? Where is it coming from? Why? |
|  | How important do you think it is to achieve the 75% OO BBV test target compared to the other priorities? |
|  | How important is it to your colleagues or manager to achieve the 75% OO BBV test target compared to the other priorities? |
|  | How will you juggle competing priorities in your own work? How will your colleagues juggle these priorities? |
|  |  |
| ***8. Organisational incentives and rewards*** | What kinds of incentives are there to help ensure that the implementation of the OO BBV testing is successful? |
|  | What is your motivation for wanting to help ensure that OO BBV testing is successful? |
|  | To what extent do you think your line manager will consider your role in OO BBV testing in your next appraisal? |
|  | Are there any special recognitions or rewards planned that are related to implementing the intervention? |
|  |  |
| ***9. Goals and feedback*** | Are changes made to healthcare clinics based on how targets and goals are being met? Can you give an example? |
|  | Do you get any feedback about the numbers of BBV tests and positive results?  How often do you get them? Where do they come from? |
|  | Do you get any feedback about the numbers of PIP seen/treated/managed for other clinical conditions? |
|  | How helpful is this/would this be? |
|  |  |
| ***10. Learning climate*** | Can you describe a recent quality improvement initiative or an implementation of a new program? |
|  | What was the motivation to improve/implement it? |
|  | What factors helped make it successful/fail? |
|  | Who were the key "players"? Roles of managers? |
|  | Were people happy with the outcome/initiative? |
|  | If you saw a patient care problem in your prison, what would you do? |
|  | To what extent do you feel like you can try new things to improve your work processes / patient care? |
|  | Do you feel like you have the time and energy to think about ways to improve things? |
|  |  |
| ***11. Readiness for Implementation*** | Assessed by the following 3 sub-constructs: |
| ***12. Leadership engagement*** | What level of endorsement or support about OO BBV testing have you seen or heard from managers? Physical health/ SMS/ head of healthcare? |
|  | How has this support affected the implementation so far? |
|  | What level of involvement has leadership at your prison / healthcare trust had so far with the intervention? |
|  | What kind of support have they given you? Can you provide specific examples? |
|  | What kind of support or actions do you need from leaders in your organization to help make implementation successful? |
|  | What types of barriers (if any) do they create? |
|  |  |
| ***13. Available resources*** | Do you have enough resources to implement enough OO BBV testing to reach the 75% target? |
|  | Are there any other resources that you would like to receive? |
|  | How do you think you should be able to obtain these? |
|  | What challenges do you expect to encounter? |
|  | How many WTE nurse vacancies are there at the moment / in the last 12 months? Any regular agency nurse use? |
| ***14. Access to knowledge and information*** | What kind of training did you receive about OO BBV testing? |
|  | Who provided the training? |
|  | Did the training leave you confident to carry out the testing / offer? |
|  | What was the most helpful aspect of the training? |
|  | Was there anything missing from the training that you would have liked? |
|  | Is there any continued training planned? |
|  | Has there been any written information about OO BBV testing made available to you?  Was it timely? Relevant? Sufficient? |
|  | Who do you ask if you have questions about the intervention or its implementation? |
|  | How available are these individuals? |
|  |  |

## D. Characteristics of Individuals

| ***D. Characteristics of Individuals (5)*** | ***Questions*** |
| --- | --- |
| ***1. Knowledge and beliefs about the interventions*** | Do you think it will be possible to achieve the 75% BBV test target in your prison?  Why or why not? |
|  | How do you feel about OO BBV testing being used in your setting? |
|  | Re: achieving the 75% target, do you have any feelings of anticipation? Stress? Enthusiasm? Why? |
|  | How do you think the OO BBV testing of 75% of PIP is going? And why? |
|  | Is the DBS test method part of OO BBV testing? |
|  |  |
| ***2. Self-efficacy*** | How confident are you about achieving 75% test rate via OO BBV approach? |
|  | What gives you that level of confidence (or lack of confidence)? |
|  | Confidence using DBS method? |
|  |  |
| ***3. Individual stage of change*** | Explore which level the individual is at using Porchaska's Stages of Change:  How prepared are you to achieve the 75% BBV testing target?  **1. Knowledge stage (Pre-contemplation)** - knowledge of key aspects of the intervention  **2. Persuasion stage (Contemplation)** - likes the intervention, discusses it with others, buys into it, has a positive view  **3. Decision stage (Preparation)** - intends to seek & try additional information  **4. Implementation stage (Action)** - acquires additional information, uses intervention regularly, and has continued use  **5. Confirmation stage (Maintenance)** - recognizes benefits, has integrated the intervention into routines, promotes use to others |
|  |  |
| ***4. Individual identification with organisation*** | Responses to other questions may be (double) coded here. For example, buy-in to organizational or intervention-related goals may be elicited under Goals & Feedback, but may also be relevant here. |
|  |  |
| ***5. Other personal attributes*** | Have you ever looked after patients with end stage liver disease such as a variceal bleed, ascites or HCC? If so, where, when? |
|  | How does that impact on your thoughts about the BBV testing targets? |

## E. Process

| 1. ***Process (9)*** | ***Questions*** |
| --- | --- |
| ***1. Planning*** | Was there a plan developed to implement the intervention? |
|  | Can you describe the plan for implementing the intervention? |
|  | Who developed the plan? Does everyone know? |
|  | Is there scope to modify or revise your plan if needed? |
|  |  |
| ***2. Engaging*** | Have you had access to knowledge / information about OO BBV testing? |
|  | How interested have you personally been about this? |
|  | How interested have other band 5 nurses / HCAs been? |
|  |  |
| ***3. Opinion leaders*** | Who are the key influencers at this prison to get on board with this implementation? |
|  | What are influential individuals saying about the intervention? |
|  | To what extent will they influence others' use of the intervention? The success of the implementation? |
|  |  |
| ***4. Formally appointed internal opinion leaders*** | Who leads the implementation of the into BBV testing?  How did they come into this role? Appointed? Volunteered? Voluntold? |
|  | What qualities does this person have that makes them a good leader of this implementation? What qualities does this person lack? |
|  | Does this person have sufficient authority to implement OO BBV? |
|  | Who else is involved with leading the implementation? |
|  |  |
| ***5. Champions*** | Are there people in your prison who champion OO BBV testing?  Can you describe people's perception of this champion/individual? |
|  | Were they formally appointed in this position, or was it an informal role? |
|  | What position do these champions have in your prison? |
|  | How do they will help with implementation? Behaviour/actions? |
|  | To what extent do you respect their opinions and actions? |
|  |  |
| ***6. External change agents*** | Did you get any outside help to implementing OO BBV testing? |
|  | Who was that, what did they do? |
|  | How helpful were they and in what ways? |
|  | How frequently do you communicate with them? |
|  | Was there a communication or education strategy for getting the word out about OO BBV testing? |
|  | Is there any on-going training / support/ feedback from PHE? |
|  | What on-going support would you like to receive? |
|  |  |
|  |  |
| ***7. Executing*** | Has OO BBV testing been implemented according to plan? |
|  | [If Yes] Can you describe this? [If No] Why not? |
|  |  |
| ***8. Reflect / evaluate*** | How do you assess progress towards OO BBV testing to 75% goals? |
|  | Do you receive feedback reports about the rate of BBV testing? |
|  | Are staff able to give feedback about what works or doesn’t work? |

## Summary Table

| **Domain** | **N= constructs** | **N= sub constructs** | **Total constructs** |
| --- | --- | --- | --- |
| 1. Intervention characteristics | 8 | 0 | 8 |
| 1. Outer setting | 4 | 0 | 4 |
| 1. Inner setting | 5 | 9 | 14 |
| 1. Characteristics of individuals | 5 | 0 | 5 |
| 1. Process | 4 | 6 | 8 |
|  |  |  | **39** |

OO BBV opt-out blood borne virus
