# Supplementary material for: An implementation science approach to determine the barriers and facilitators to hepatitis C virus testing in English remand prisons: a mixed-methods study
Source: BMJ Open. 2025 Oct 29;15(10):e092965. doi: 10.1136/bmjopen-2024-092965 (PMC12574346; doi:10.1136/bmjopen-2024-092965)
Supplement: online supplemental file 5 [file bmjopen-15-10-s005.docx]

**Complete data with illustrative quotes**

#### Table 4. Consolidated Framework for Implementation Research (CFIR) Barriers to implementing anti-HCV testing in prisons

| **CFIR domain and construct** | **Construct descriptor** | **Summary of responses** | **Illustrative quote(s) and participant ID number.** |
| --- | --- | --- | --- |
| **A1** | Knowledge about intervention source | Only one participant knew about the background to opt-out testing | "I can't remember the circumstances around it…I couldn’t tell you the full background" #8; "I don’t know who decided to have the opt-out, which seems like not the best decision" #9 |
| **A2** | Strength and quality of Intervention evidence | None aware of any research evidence behind the opt-out approach. Introduced "top down" | "There was a public health strategy I believe" #10. |
| **A5** | Was the intervention piloted? | Not piloted in all prisons to identify local issues | "The problem is it's been mixed in with this you’ve got to do it in your first reception"….and we have meeting after meeting after meeting about it and trying to make people realise that each site isn't the same and doesn’t have the same facilities to do everything" #3 |
| **A6** | Intervention complexity and understanding definition | Opt-out testing and DBST testing considered to be the same by the majority, most responses focussed on the concept of choice. | "At the end of the day they still get the choice, it’s a medical intervention" #7; "I guess offering it to every single person and then they can choose not to have it, making it more of an option" #13 |
| **A7** | Intervention packaging/training | Initial training delivered by written material, not in person ,agency nurses not included, training focused on the DBST not opt-out approach | "Most of the focus of the training was about the equipment rather than the approach. We put together a guide for staff in terms of changes in the questions, rather than the actual DBST testing which did have training attached to it" #10 |
| **B1** | Knowledge of the patient's needs and resources | When people first enter a prison, the reception process is too busy for the PIP to think about a HCV test, especially in a remand prison. Some PIP feel they don’t need a test because they have never injected drugs, some fear being found to be HCV positive so decline a test, testing fatigue occurs with repeated testing for frequent reoffenders, and some PIP find it hard to trust people including nurses. | "He's been in police cells the night before, been in court all day, withdrawing off his drugs, by the time he makes it through to healthcare at about 6 or 7 o'clock in the evening he's got no patience left. He just wants to get it done, whatever the questions are, and go and get his medication" #7; "People are sick of being offered the same thing over and over again...our population group often feel quite saturated " #10; "When I ask about hepatitis a lot of them just shrug their shoulders and just say, 'I don't know...I can't be bothered', so the disinterest is palpable" #11 |
| **B2** | Cosmopolitan - opportunities to meet other nurses to discuss the intervention | Registered nurses don’t get the chance to meet nurses from other prisons in the region or nationally to share best practice and problem solve unless they are a matron (a managerial role), and attendance can be determined by staffing levels. Liaising with hospital specialist nurses is very helpful. | "I've only been to one of the matron's days" #2 “We had quarterly forums (with hepatitis operational delivery network …evidence it was reducing hep C patients and how the change in our approach had increased the ODN's treatment by 120%" #4 |
| **B3** | Peer pressure - how this can influence opt-out HCV testing | One participant paraphrased negative comments overheard about the value of opt-out HCV testing in prisons. These views constitute negative peer pressure that could discourage testing. | "I've heard some really bad things in other establishments and what their views are around this [opt-out HCV testing], like 'oh they're [PIP] not here long enough, we don't need to bother' and 'what’s the point, they're [PIP] not going to get treatment' " #6 |
| **B4** | External policies and incentives | Linked to A1 and A2. Participants not aware of the policy to test 75% of PIP on arrival. Nationally written prison regime policies are enforced robustly by prison officers which impacts on opportunities for healthcare provision. NHSE/ public health policy for opt-out HCV testing criticised because "outsiders" don't understand "prison life". | "The prison has the 'bus to bed' time frame, once the prisoner arrives they have to get them completed through the reception process and to their cell. There's a lot of people that the prisoner has to see when they arrive in reception and if they breach that process, I'm not exactly sure what the consequences are but there is, ...healthcare is the last person to see the prisoner so that limits the amount of time that you have to see each patient, and in a remand you can be getting up to 25 patients through in an afternoon, so it limits how much time you have to complete each section of the reception screen ...so that adds additional pressure to what interventions you can actually physically do when you're in prison" #13 |
| **C1** | Structural characteristics | The lack of space in the reception area and on the wings for healthcare provision was reported by most participants. Some rooms were structurally dilapidated. Most nurses identified there was insufficient space to do testing. | "It's like a little shoebox that healthcare have been shoved into and it's not fit for purpose. It doesn’t have a sink" #13 "We could do with a clinic room on each wing. There's one on (name of) wing but that's condemned" #12 " The facilities here are antique" #9 |
| **C2** | Networks and communications | Prisons are hierarchical organisations. Healthcare roles don’t overlap e.g. Substance misuse, mental health, and primary care nurses all have very distinct remits. Successes are dependent on working relationships with prison staff. | "A lot of things [patient care] do overlap but have been told 'you're to stick to your own lane' " #8 "The physical care side believe it's more of a substance misuse issue so there's resistance...it's childish at times" #8 "Some prison officers see healthcare as an inconvenience" #5 |
| **C3** | Work culture | Many prison officers are perceived to be uncaring, and not all are supportive of preventative healthcare activity. There was some evidence of a poor work culture among the nurses too. | "We're being pushed by prison staff because they need to take the PIP down to the wings and lock up, so they keep pushing; 'are you ready to see the next one 'cause they need locking up?' So you get that side of stress as well" #7 "I’ve witnessed staff (nurses) that are absolutely abysmal, they’re just like ' no he doesn’t want it', and it's just not opt-out. I think it gives them a get out of jail card" #9 |
| **C4** | Implementation Climate | Negativity was identified amongst some participants, with low staff morale and a reluctance to work observed in some colleagues. This results in some nurses being uninterested in HCV and all BBV testing and a view held by some that that there is no point in testing because it takes too long to get results back. | "The view of some nurses is 'so why would you want to give yourself more work by saying "hey, now we're going to do this" (DBST)" #4 "I don't think that staff themselves who are testing believe that it is as important as we all think it is. I Think that’s where the problem is...if other prisons can do it and we can't, then obviously there's a problem with our particular prison". #11 |
| **C7** | Relative priority of the intervention compared to others | The majority of participants' comments were in this domain. The priority in reception is to get the PIP in and processed, assess for medicines needed and acute issues to respond to for their first night, to assess for suicide risk or self-harm and avoid deaths. Other healthcare priorities include: wound care/dressings, emergency responses, cancers, long term conditions, vaccinations e.g. flu/Covid. During their sentence PIP may be more interested in addressing other health issues e.g. dental care, and may also be concerned with managing threats from gang members, bullying, being a vulnerable prisoner. The prison regime is a significant competing priority. | "Prison officers say; 'we haven't got time for this….we need to move you, get you moved to the wings'" and "If someone works in the kitchens, the officer will go and collect them, kitchen staff may say " do you really need to go? We could really do with you here" #1 "So they're frustrated, they might be hungry, they're tired, they want a cigarette, they want to get onto the wing to get a vape. They want medication, they're hungry and want a shower, they're tired, they're upset. They've been sentenced or got a longer sentence than expected, or they're upset because they think they’re not guilty and they've been sent to prison”. #7 "You know how long an admission takes to do in a hospital? Imagine having 20 at the same time". #7 |
| **C9** | Goals and feedback | There is limited feedback given to nurses about process or achievements, numbers tested or the outcomes. Sometimes feedback about low test uptake is used punitively with limited recognition that PIP refuse tests. | "I think it's [feedback data] absolutely nonsense because you can't compare [the uptake percentages of] a small population of 500 men in a training prison who aren't going anywhere to a revolving belt of a larger remand prison" #5 |
| **C12** | Leadership engagement | Prison leadership: healthcare staff are keen to make changes but there can be resistance from some prison officers because it can mean extra work for POs to escort PIP to healthcare. Prison Governors can block HCV Trust peer worker access. Healthcare leadership: Limited or sporadic encouragement to achieve higher rates of anti-HCV testing from senior leadership teams was observed by a minority of participants. | "If you get some of the more reliable officers, you'll probably get most of the patients that are down on your clinic list, but more often than not we are moaning to the matron and head of healthcare because certain officers won't be able to 'find' them and they tell us 'oh yeah he refused', but we get frequent feedback from the patients that they haven't refused the appointments. It's so frustrating" #12 "There's plenty of pressure applied for the vaccines, but not so much for the DBSTs....every now and then you do hear from the management team that we need to increase the amount of DBSTs we're doing and there'll be a little bit of a push for them, but from what I've witnessed it's minimal pressure" #12 |
| **C13** | Available resources | Agency nurses make up the number of staff needed each shift but they cannot lead implementation projects. There are insufficient nurses, and it is difficult to recruit nurses to work in a prison in the context of a national shortage. There are often insufficient prison officers too and this combined lack of staff is a significant resource gap. | "you hear that there is money for this and that, but money is useless if you cannot have a body that you pay to do the job" #5 |
| **D2** | Staff self-efficacy | Individual staff self-efficacy is variable. A few participants report that some nurses are apathetic towards HCV testing and that this is generally driven by staff who are interested rather than it being an embedded process. | view of some nurses; "so why would you want to give yourself more work by saying "hey, now we're going to do this" (DBS test)" #4 “I think the prisoners are apathetic, and the staff are apathetic, and that is a pretty depressing combination" #11. |
| **D5** | Other personal attributes | Most prison nurses won't have clinical experience of looking after people with liver disease. However, one participant had looked after people with decompensated cirrhosis. | "The variceal bleeds were always messy, to the point where a mop and broom just wouldn't handle it" #12 |
| **E4** | Formally appointed Internal opinion leaders | Unlike the prisons whose healthcare services are run by Patient Partnership Group, there are no formally appointed leaders employed by the three prisons. This absence of a key individual to lead this aspect of service delivery was observed by a minority of the participants. | "We could do with a dedicated in-house lead to really push it, like a leafleting campaign, regular workshops with the patients, support groups and one to ones" #12 |
| **E5** | Champions | Champions may be officially appointed, such as the national group of hepatitis C Trust employed peer workers who are able to support education and testing. Other Champions may be current PIP who are influential among their peers and willing to support others. However, PIP don't always engage with Champions because of trust and confidentiality, and they may have an ulterior motive for seeking a role where they can move more freely in the prison. Hard to find the right person to be a Champion in a cat B because of the high turnover. One prison would like to have HCV Trust prison peer workers, but the Governor has refused. | "They're [the Champion] probably wheeling and dealing but they're the 'go to' person in the prison" #6 " no-one wants to say to the prisoner (champion) 'oh I'm on hep C meds' because they'll get beaten up or something" and they don’t want to get into trouble with other PIP… who can you trust?" #4 |

#### Table 5. Consolidated Framework for Implementation Research (CFIR) Facilitators to implementing anti-HCV testing in prisons

| **CFIR domain and construct** | **Construct descriptor** | **Summary of responses** | **Illustrative quote(s) and participant ID number.** |
| --- | --- | --- | --- |
| A4 | Adaptability- changes made or suggested to improve test uptake | Changing the timing to subsequent healthcare appointments, extend substance misuse nurses' remit to test, enable prison and community electronic health records to be compatible to aid communication. Prison officers to tell the PIP that testing is routine. | "Rather than being done at first reception in most establishments, is now being completed in the second health screen which is within the 72 hours of arrival" #10. "Prison officers to say that 'every man who comes into the establishment must be tested for BBVs' #5. "We (drugs workers) would really like to do the testing....I think it would be easier for us to be able to catch people for testing and we've probably got more availability to do so, but that’s not up to us, that’s the commissioners" #1. |
| B2 | Cosmopolitan - opportunities to meet other nurses to discuss the intervention | Matrons (band 7s) meet regularly with other band 7s in their region, and there are good links with hospital HCV nurses who share information and support. It would be helpful for the staff nurses to have external peer support too. | "Would be really good to actually spend time with people in similar situations because it does feel a little bit that you’re on your own, it’s such a niche place to work" (nurse wanting to meet other prison nurses) #1 |
| C1 | Structural characteristics | Newer prisons have purpose-built healthcare space which facilitates improved care delivery. | "The newer ones are more purpose built…there's a shift towards acknowledging that these kinds of environmental factors play a big part in what we do" #10. |
| C8 | Incentives and rewards | Incentivising the PIP is highly effective, e.g. shopping vouchers to have on release, phone credit, puzzle books, extra gym visits. Financial incentives for nurses to work in prisons was suggested. | "Extra gym sessions because they may not have all the gym sessions that they would like, and we've been supportive of that one because it's a healthy thing" #3. "[other NHS environments] pay extra money on top of peoples' (nurses) salaries 'cause of recruitment and retention [difficulties] " #6 |
| C9 | Goals and feedback | Seeing people cured is motivating. Important to give feedback about test uptake that recognises different prison contexts. | "They compare the uptake percentages of sentenced prisons to remand prisons, to me it's utter nonsense and stupid" #5. |
| C14 | Access to knowledge and information | Increase provision of teaching nurses about the opt-out approach, test methods, and HCV (and other BBVs). Include as part of the formal staff induction process. | "I could do with knowing more about it but it's just not something that's offered to us... maybe part of the induction package when you get new starters" #8 |
| E1 | Planning for intervention implementation | External support from the NHS Trust's Quality Improvement team using the Plan, Do, See, Act (PDSA) methodology. Nurses monitored the day-to-day testing data (same as PPG). | "We had support from the Quality Improvement Team who were helping us navigate some of the methodology, the PDSA cycles" #10 |
| E4 | Formally appointed Internal opinion leaders | Noted by the research team that the prison healthcare departments run by the Patient Practice Group also employ a regional HCV lead nurse to oversee the HCV testing programme. The authors and one participant suggest that a similar NHS role would be advantageous in all category B local prisons. | "We need to nominate dedicated staff for it, that means extra funding" #12 |
| E6 | External Change Agents | HCV Trust employed prison peer workers are valued. Outside help from Hep C Trust who deliver the High Intensity Test and Treat (HITTs) events is welcomed. | "They (HITTs) are helpful…physically having people coming in and supporting, it's boosted morale for the team, raising the profile of it, and the fact that you're getting so many done in one day, is a good sense of achievement isn't it?" #3 |
