# Supplementary material for: An implementation science approach to determine the barriers and facilitators to hepatitis C virus testing in English remand prisons: a mixed-methods study
Source: BMJ Open. 2025 Oct 29;15(10):e092965. doi: 10.1136/bmjopen-2024-092965 (PMC12574346; doi:10.1136/bmjopen-2024-092965)
Supplement: Supplementary file 6 [file bmjopen-2024-092965.reviewer_comments.pdf]

PEER REVIEW HISTORY

BMJ Open publishes all reviews undertaken for accepted manuscripts. Reviewers are asked to complete a checklist review form and are provided with free text boxes to elaborate on their assessment. These free text comments are reproduced below.

ARTICLE DETAILS

Title (Provisional)

An implementation science approach to determine the barriers and facilitators to hepatitis C virus testing in English remand prisons: A mixed methods study.

Authors

Jack, Kathryn; Irving, William; Rose, Zoe; Thomson, Brian James

---

VERSION 1 - REVIEW

**Reviewer**	**1**
**Name**	**Lim, Beng Leong**
**Affiliation**	**National University Health System, Emergency**
**Date**	**24-Sep-2024**
**COI**	**None**

I thank the editor for asking me to review this manuscript. This is a mixed quantitative and qualitative study using data sets, questionnaire and structured interviews to investigate the facilitating reasons and barriers to Hepatitis C testing in 3 category B prisons in England.

The methods are logical and sound and the conclusions valid. There are some clarifications and suggestions to improve this manuscript.

General

1. I think the abbreviations like HCV, HBV and PIP should be explained at first use and then the abbreviations put in brackets.

2. I think the authors should give a brief description of what is a Category B prison? This concept might not be familiar for international readers and might have impact on better understanding of the study design. Are Category B prisons for what type of crimes, for short-term or long-term sentences, etc?

Methods

1. I think the authors should explain why the 3 prisons were selected and how representative of the "problematic prisons" with the lowest HCV testing?

2. I think the authors should explicitly state the inclusion and exclusion criteria of the study? Are they only recruiting new entrants to these 3 prisons or all people in prison (PIP); either existing or new in these 3 centres? If they are only recruiting new entrants, why they choose to exclude existing PIP? The existing PIPs might also have low rates of HCV testing and their rates might have decline with length of stay.

3. For the questionnaire part to investigate the rationale for anti-HCV test refusal, I think it is important to state what is the overall total estimated number of PIP who refused testing in these 3 centres. Is 166 PIP the overall total or close to it or just a fraction of it? How many of the total PIPs who refused testing decline to participate in this survey? This will allow the readers to have an idea whether the 166 participants who took part in the questionnaire are truly reflective or there is selection bias.

4. I think it is important to state briefly how the questionnaire instrument is constructed and what areas or domains of facilitating reasons or barriers did it encompass?

5. How was the questionnaire delivered? Was there chance for participants to interact with each other or is it conducted independently?

6. What was the rate of incomplete answers in the questionnaire? Was it a significant portion that might affect the interpretation of the results?

Reviewer **2**

Name **Ali, Salamat**

Affiliation **Quaid-i- Azam University, Islamabad , Department of Pharmacy,**

Date **05-Nov-2024**

COI **None**

Overall, the research paper presents valuable insights and contributes meaningfully to the field. However, a few minor revisions could enhance its clarity and impact which are; there seems to be some ambiguity between title and stated objectives , in the methodology section add more details of questionnaire, in the findings section, Table 2 would benefit from further explanation to help readers interpret the data more effectively.

**** The reviewer provided a marked copy with additional comments. Please contact the publisher for full details. ****

Reviewer **3**

Name **Akiyama, Matthew J.**

Affiliation **Albert Einstein College of Medicine**

Date **08-Apr-2025**

COI None

This study highlights an important topic on systems level challenges on HCV testing uptake in remand prison settings in the UK.

Abstract:

Presumably these %'s in the abstract represent the 3 prisons mentioned in the first sentence of the Conclusions but this should be clarified. "17.2%, 28.3% and 42.5%."

Page 6

Line 48 - PIP should be spelled out at first instance

Page 7

Line 8 - The source of this definition of microelimination should be mentioned, which is specific to the great work of The Hepatitis C Trust, but is not universally done.

Page 8

Line 44 - Why was length of stay and prior location only collected for the specified amount of time? A rationale should be provided.

Line 57 - How the 166 participants sampled? The methods for doing so should be specified.

Page 10

Line 26 - The analysis would be strengthened by reporting the methods used in line with COREQ criteria and providing the COREQ checklist as an addendum.

Line 41 - What proportion of the sample was length of stay and prior location available for? Why was this only available for a subset?

Page 11

Line 17 - This acronym should be spelled out.

Page 12

Line 42 - "there are 21 are" needs to be revised.

Page 14-16 - It would be more straightforward to use the same notation as the CFIR website e.g. Domain I, II, etc. Constructs A, B, etc.

Page 17

Line 47 - Would state the CFIR domain rather than just "Domain D". E.g. Domain D: Characteristics of Individuals.

Line 55 - This high level summary of results belongs in the results section -- e.g. Page 12 line 44.

Page 21

Line 18 - Additional limitations should include the partial sampling, which should be explained further as outlined above.

**Reviewer**	**4**
**Name**	**Jean-Louis, Michtta**
**Affiliation**	**Centers for Disease Control and Prevention**
**Date**	**24-Jul-2025**
**COI**	**None**

Summary

- Author presented the information in an orderly fashion
- Author appears to address a research gap and global issue that may support future public health interventions for the sub-population
- Author described limitations of the paper
- Author addressed potential next steps for overcoming barriers to anti-HCV test uptake
- Paper would benefit from slight revision in regards to sentence structure and flow.
- Author used acronyms that were not adequately defined early on in the paper such as PIP, NHS, CFIR, HCV
-

Major:

- Consider adding inclusion and exclusion criteria's in the methods section of paper (page 7) for why the specific prisons were selected, did you exclude incarcerated persons or was everyone included in the analysis.
- Consider explaining why
- To improve clarity of the paper, suggest revising sentence structure and flow through out paper.

Minor:

- Consider adding population size of the region to the setting section (page 3)
- Consider spelling out acronym prior to introducing it in the paper. For example: HCV, PIP, NHS, CFIR, HMPPS
- Since this paper will reach an international audience, consider explaining the structure of a category B reprimand prison and why it was selected over other types of correctional facilities in the area.

- Not sure if it's a difference in regional usage but it is unclear what $2p<.001$ means? Usually it is written as $p<0.001$. Consider checking to ensure the statistic format is correct (page 10 and 11)
- On page 6 paragraph 3 sentence 1 "Ongoing HCV transmission in the UK continues via sharing contaminated illicit drug injecting paraphernalia, so the prison estate.....". Is this sentence stating that injection drug use is one of the highest reasons for prison reprimand or is the risk behavior high overall in the UK. Consider revising this sentence for clarity
- Data available for full 52 weeks for anti-HCV test uptake however variable date timeframes available for length of stay and prior location. Consider elaborating as to why this discrepancy might be noted. (page 10)
- Consider expanding table title to include date of data collection. For example, table 1 (page 11), "Table 1. Length of stay and anti-HCV test uptake n=1836 (Month, Date, Year – Month, Date, Year)

VERSION 1 - AUTHOR RESPONSE

Reviewer 1	Authors' responses	Page
I think the abbreviations like HCV, HBV and PIP should be explained at first use and then the abbreviations put in brackets.	The abbreviation HCV is explained at first use in the background to the Abstract. The abbreviation PIP is explained at first use in Results section of the Abstract. The abbreviation HBV is explained at first use in Background section of the main text.	2<br>3<br>5
I think the authors should give a brief description of what is a Category B prison? This concept might not be familiar for international readers and might have impact on better understanding of the study design. Are Category B prisons for what type of crimes, for short-term or long-term sentences, etc?	We have added the statement "which typically accommodate men who are awaiting sentencing, serving a very short sentence, or nearing the completion of a long sentence and are being prepared for release in their home town".	6
1. I think the authors should explain why the 3 prisons were selected and how representative of the "problematic prisons" with the lowest HCV testing?	We have added the following section:<br>Participating Sites<br>The three sites were selected from the complement of 30 Category B Remand prisons in England and Wales. All the selected sites are in the East Midlands of England, which is the same geographic location as the authors' place of work and all have their healthcare managed by the NHS. Further, the three prisons selected contained sites representative of both historically low and historically high testing rates.	7

2. I think the authors should explicitly state the inclusion and exclusion criteria of the study? Are they only recruiting new entrants to these 3 prisons or all people in prison (PIP); either existing or new in these 3 centres? If they are only recruiting new entrants, why they choose to exclude existing PIP? The existing PIPs might also have low rates of HCV testing and their rates might have decline with length of stay.	We have added the following sentences:<br>The primary purpose of our study was to assess progress towards the national target of testing 75% of new prison entrants within a two-week period and the factors which impact on these testing rates. In order to address whether testing of entrants after this period could increase numbers tested we elected to extend our data collection for a total of four weeks. Known HCV-positivity in PIP who were already inmates was collated but was not linked to date of testing.	7
3. For the questionnaire part to investigate the rationale for anti-HCV test refusal, I think it is important to state what is the overall total estimated number of PIP who refused testing in these 3 centres. Is 166 PIP the overall total or close to it or just a fraction of it? How many of the total PIPs who refused testing decline to participate in this survey? This will allow the readers to have an idea whether the 166 participants who took part in the questionnaire are truly reflective or there is selection bias.	The text has been revised: Reasons for anti-HCV test refusal were obtained via the questionnaire during a 43-week period between 21st March 2022 and 15th January 2023. There were n=5,469 people admitted to the three prisons in that time, of whom only 25.73% (n=1407/5469) were tested for anti-HCV. The number of PIP not tested therefore vastly exceeded the study teams' predictions which had been based on the available literature at the time of designing the study. It was beyond the capacity of the study to establish the reasons why n=4062 PIP refused a test. Data on the reasons for not being tested were sampled using an unselected group of n=166 PIP and are set out in Table 2. The principal limitation on this data collection was constraints on the availability and time of prison nurses. using an unselected and convenience sample of n=166 PIP, see Table 2.	12
4. I think it is important to state briefly how the questionnaire instrument is constructed and what areas or domains of facilitating reasons or barriers did it encompass?	Additions have been made to the text:<br>The questionnaire was designed by the study team and comprised of three groups of questions: firstly to ascertain if there was a medical reason why it was not appropriate to offer a test, such as a diagnosis of dementia or acute psychosis; secondly to ascertain the PIP's reasons for refusal which included: a belief they were not at risk; having been tested recently elsewhere; already known to be HCV positive; needle phobia; not interested; had never taken intravenous drugs; asked why they should be tested. The third section was to record if there had been any prison security regime related reason that would have prevented PIP from accessing a routine healthcare appointment to be tested.	9

5. How was the questionnaire delivered? Was there chance for participants to interact with each other or is it conducted independently?	The following text has been added: Questionnaires were administered by prison nurses during the clinic appointment when PIPs were offered a test. The nurse and PIP worked together to ensure that all applicable boxes were ticked, ensuring a very high completion rate of the questionnaire. Successful completion of this study aim was also dependent on availability of prison staff and time within the prison security regime.	9
6. What was the rate of incomplete answers in the questionnaire? Was it a significant portion that might affect the interpretation of the results?	See answer to 5. above	9
**Reviewer 2 (comments in PDF of the proof)**	**Authors' responses**	**Page**
Ambiguity between title and selected objectives	Selected objectives have been modified to resolve any ambiguities with the title.	2
Needs explanation regarding questionnaire (CFIR)	See Comments to Editor	10
Last sentence: explanation needed	The last sentence has been altered to improve clarity as follows:<br>Finally, it is clear that when PIP are presented with the ability to opt-out of a healthcare intervention, a substantial number do so. It may therefore be timely to modify the approach to prison-based HCV testing and reframe the current testing intervention as "routine" rather "opt-out" as this may minimise future uncertainty regarding the importance of ongoing testing for HCV in prisons.	24
Facilitators explanation is satisfactory		18
Conclusion not properly explained according to title and objectives	We have revised this section of the Conclusion to more clearly describe our findings and to better align with the newly restated objectives:<br><br>Our study has confirmed previous concerns that anti-HCV test uptake in some male category B remand prisons managed by the NHS remains far short of national standards. Our study operated in an environment in which the availability of prison staff key in both facilitating our study and delivering healthcare was often unavoidably compromised. This enabled us to	24

	unpack explanatory findings which were contextual and which identified barriers and facilitators to implementing the opt-out testing intervention in the study prison environment . We have three key recommendations to enable embedding a sustained process to anti-HCV testing and reducing the negative impact of the barriers. Firstly, the appointment of a dedicated senior healthcare staff member who has the overall responsibility, accountability, and authority to proactively oversee testing and ongoing referral processes. Secondly, to reintroduce an education programme for prison healthcare teams to teach about HCV, cirrhosis, and how to deliver "opt-out" conversations and respond to typical responses. Third, for NHS and Crime and Justice leaders to negotiate reforms to the prison regime to enable the delivery of contemporary healthcare needs for the prison population.	
**Reviewer 3**	**Authors' Responses**	**Page**
Abstract:<br>Presumably these %'s in the abstract represent the 3 prisons mentioned in the first sentence of the Conclusions but this should be clarified. "17.2%, 28.3% and 42.5%."	The sentence has been re-written: The Prison testing rates within 4 weeks of arrival in the three category B male prisons…"	3
Page 6<br>Line 48 - PIP should be spelled out at first instance	This has been corrected. (People in Prison).	6
Page 7<br>Line 8 - The source of this definition of microelimination should be mentioned, which is specific to the great work of The Hepatitis C Trust, but is not universally done.	Reference added: Lazarus et al (2018), reference number 6.	7
Page 8<br>Line 44 - Why was length of stay and prior location only collected for the specified amount of time? A rationale should be provided.	The length of stay and prior location is held on the electronic prison management system and can legally only be accessed by prison employees. This data was therefore obtained by the prison nurses. The number of prison entrants and test uptake data were obtained for 52 weeks. Length of stay and prior location metrics were collected from Prison #1 for 3 weeks. , Prison #2 for 28 weeks, and Prison #3 for 28 weeks – a total of 59 prison weeks. As noted above, access to these variables is wholly dependent on the availability of prison staff and	8

	the prison security regime, which unavoidably limited data collection.	
Page 8 Line 57 - How the 166 participants sampled? The methods for doing so should be specified.	We are unclear whether this question refers to i) the administration of the questionnaire to the 166 participants or ii) the methods for selecting a subset of 166 participants.<br><br>If i) please see response to reviewer 1 question 5<br><br>If ii) we have introduced the following statement: ‘Successful completion of this study aim was also dependent on availability of prison staff and time within the prison security regime’. Please also note a new sentence added to page 19 of the Discussion in reference to why only 166 PIP were sampled. ‘This is likely to be a consequence of pressures on staff time and security requirements for an exercise that required additional nurse time to complete.’ (therefore the subset of PIPs recruited was determined solely by variable staffing levels and not on any characteristics of the PIP likely to lead to led to selection bias).	9
Page 10<br>Line 26 - The analysis would be strengthened by reporting the methods used in line with COREQ criteria and providing the COREQ checklist as an addendum.	COREQ qualitative reporting checklist provided as a supplementary file.	
Line 41 - What proportion of the sample was length of stay and prior location available for?<br><br>Why was this only available for a subset?	Test amended to read: “Data describing PIPs’ length of stay and prior location were available for 28 weeks from Prisons #2 and #3 individually (53.8% of the test uptake timeframe), and for three weeks from Prison #1 (1.6% of the test uptake timeframe)”<br><br>For response to the second part of the question see answer to Page 8 line 44 question above. Additional text: “This data could legally only be obtained by nurses employed by the prisons and data collection was impacted by staff availability at each testing site”	11
Page 11<br>Line 17 - This acronym should be spelled out.	The acronym UKHSA explained in full (United Kingdom Health Security Agency)	11

Page 13<br>Line 42 - "there are 21 are" needs to be revised.	Changed to "there are 21"	13
Page 14-16 - It would be more straightforward to use the same notation as the CFIR website e.g. Domain I, II, etc. Constructs A, B, etc.	Notation changed in Tables 3, 4 and 5.	14-16
Page 17<br>Line 47 - Would state the CFIR domain rather than just "Domain D". E.g. Domain D: Characteristics of Individuals.	Text changed to:<br>in domain IV: Characteristics of Individuals, and notation altered in the discussion	18<br><br>20-22
Line 55 - This high level summary of results belongs in the results section -- e.g. Page 12 line 44.	The following text moved to the results section: "The healthcare staff who participated in the qualitative semi-structured interviews identified a number of barriers and facilitators to the achievement of high levels of HCV testing in this setting that can be summarised in these five overarching themes: misunderstanding of the concept of opt-out testing; nurses not meeting performance targets due to competing priorities; prison regime hampering healthcare delivery; absence of a specifically appointed co-ordinator who is held to account; incentivising nurses to test and PIP to accept testing"	55 and 13
Page 21<br>Line 18 - Additional limitations should include the partial sampling, which should be explained further as outlined above.	We have added the following sentences to this section; The key limitation throughout our study was that non-prison staff are not permitted to make direct contact with prisoners or access data kept on prison information systems. Many key aspects of our research, and the success of the prison healthcare systems being studied, are a function of the time and availability of the prison staff, which were often and unavoidably compromised.	23
**Reviewer 4**	**Authors' Responses**	**Page**
Paper would benefit from slight revision in regards to sentence structure and flow.	We have reviewed the entire text and introduced revisions to improve clarity, focus and flow as best we can.	
Author used acronyms that were not adequately defined early on in the paper such as PIP, NHS, CFIR, HCV	HBV and HCV are first explained in the background section.<br>An explanation of HCV has been added to the abstract.<br>CFIR is first explained in the abstract and in the regulatory approval sections.<br>NHS and HMPPS explanations added.	6<br><br>2<br><br>2,9<br><br>4

Consider adding inclusion and exclusion criteria's in the methods section of paper (page 7) for why the specific prisons were selected, did you exclude incarcerated persons or was everyone included in the analysis.	See response to reviewer 1.	7
Consider explaining why…		
To improve clarity of the paper, suggest revising sentence structure and flow through out paper.	See answer above	
Consider adding population size of the region to the setting section (page 3)	The three selected prison serve a combined population of 2.3 million and have the capacity to accommodate a total of 2030 prisoners.	
Consider spelling out acronym prior to introducing it in the paper. For example: HCV, PIP, NHS, CFIR, HMPPS	Thank you for this observation. HBV and HCV are first explained in the background section.<br>An explanation of HCV has been added to the abstract.<br>CFIR is first explained in the abstract and in the regulatory approval sections.<br>NHS and HMPPS explanations added.	6<br>2<br>2,9<br>4
Since this paper will reach an international audience, consider explaining the structure of a category B reprimand prison and why it was selected over other types of correctional facilities in the area	Please see response to reviewer 1.	
Not sure if it's a difference in regional usage but it is unclear what 2p<.001 means? Usually it is written as p<.001. Consider checking to ensure the statistic format is correct (page 10 and 11)	This is a typo and has been corrected.	10-11
On page 5 paragraph 3 sentence 1 "Ongoing HCV transmission in the UK continues via sharing contaminated illicit drug injecting paraphernalia, so the prison estate…..". Is this sentence stating that injection drug use is one of the highest reasons for prison reprimand or is the risk behaviour high overall in the UK. Consider revising this sentence for clarity	This sentence has been re-written as: The predominant risk factor for ongoing HCV transmission in the UK is the sharing of contaminated illicit drug injecting paraphernalia. This activity is prevalent in UK prisons and consequently the prison estate harbours a higher proportion of HVC positive individuals than the general community. (reference follows in the text)	5
Data available for full 52 weeks for anti-HCV test uptake however variable date timeframes available for length of stay and prior location. Consider elaborating as to why this discrepancy might be noted. (page 10)	See response to reviewer 3 (page 8, line 44)	10

Consider expanding table title to include date of data collection. For example, table 1 (page 11), "Table 1. Length of stay and anti-HCV test uptake n=1836 (Month, Date, Year – Month, Date, Year)	We have changed the tittle of Table 1 to: "Table 1. Length of stay and anti-HCV test uptake n=1836 (data collected in prison #1 for 3 weeks and in prisons #2 and #3 for 28 weeks duration between March 03 2022 to September 26 2022)".	11

VERSION 2 - REVIEW

Reviewer 1

Name Lim, Beng Leong

Affiliation National University Health System, Emergency

Date 03-Sep-2025

COI

The authors have addressed my concerns in this revision; specifically on how the interview questionnaire was constructed, insights into how many people in prison decline testing as well as why the 3 study centres were selected in the study.

Reviewer 2

Name Ali, Salamat

Affiliation Quaid-i- Azam University, Islamabad , Department of Pharmacy,

Date 18-Sep-2025

COI

Appreciate the novel work undertaken by authors. It is indeed a prevailing concern on the data fron prison estate. There are few suggested changes required to be inclrporated in the MS during minor revision;

Firstly, the recommendations need to revised because much of its text is not proved from the data of this research i.e for policy makers and healthcare system.

Secondly, the tables should define all abbreviations in the foot notes.

It is encouraging to see STROBE checklist has been followed for reporting this study. However, a mixed methods study may be reported as mix of Equators checklist such as for observational and qualitative studies are undertaken and relevant checklist should be preferred.

VERSION 2 - AUTHOR RESPONSE

Reviewer	Authors' responses	Page
Firstly, the recommendations need to be revised because much of its text is not proved from the data of this research i.e. for policy makers and healthcare system.	Thank you for this advice. We consider that the first two recommendations are amply supported by our findings but agree with your reviewer that the third, for the NHS and Crime and Justice leaders, is not sufficiently evidence based. We have therefore replaced this with the alternative: 'Thirdly to adopt more widely the strategy already shown to be successful in increasing test uptake by the Hepatitis C Trust HITT programme and offer prison nurses and PIP simple incentives such as confectionary.' This is a pragmatic proposal which was flagged up as a potential key facilitator in our analysis of the CFIR.	23
Secondly, the tables should define all abbreviations in the foot notes.	Thank you for this observation. The abbreviations used in Tables 1,2, 4 and 5 have been explained underneath.	12, 13, 16,17
It is encouraging to see STROBE checklist has been followed for reporting this study. However, a mixed methods study may be reported as mix of Equators checklist such as for observational and qualitative studies are undertaken and relevant checklist should be preferred.	I have already submitted two Equator reporting guidelines checklists;<br>Supplementary file 3: **COREQ** (for **qualitative** research)<br>Supplementary file 4: **STROBE** (for **observational studies**).<br>I have included both of these in this resubmission in case they were not previously sent to you.	
If you have selected 'Yes' above, please provide details of any competing interests.: Not applicable	Competing interests statement added to manuscript and the submissions portal.	24
